# Quantifying cognitive and affective impacts of Quizlet on learning outcomes: a systematic review and comprehensive meta-analysis

**DOI:** 10.3389/fpsyg.2024.1349835

**Published:** 2024-03-06

**Authors:** Osman Özdemir, Hümset Seçkin

**Affiliations:** ^1^Foreign Language Education, School of Foreign Languages, Selcuk University, Konya, Türkiye; ^2^Foreign Language Education, School of Foreign Languages, Akdeniz University, Antalya, Türkiye

**Keywords:** Quizlet, attitude, retention, achievement, meta-analysis, vocabulary learning, mobile assisted learning

## Abstract

**Background:**

This study synthesizes research on the impact of Quizlet on learners’ vocabulary learning achievement, retention, and attitude. Quizlet’s implementation in language education is posited to enhance the learning experience by facilitating the efficient and engaging assimilation of new linguistic concepts. The study aims to determine the extent to which Quizlet influences vocabulary learning achievement, retention, and attitude.

**Methods:**

Employing a meta-analysis approach, this study investigates the primary research question: “Does Quizlet affect students’ vocabulary learning achievement, learning retention, and attitude?” Data were collected from various databases, identifying 94 studies, of which 23 met the inclusion criteria. The coding reliability was established at 98%, indicating a high degree of agreement among experts. A combination of random and fixed effects models was used to analyze the effect size of Quizlet on each outcome variable.

**Results:**

Quizlet was found to have a statistically significant impact on learners’ vocabulary learning achievement, retention, and attitude. Specifically, it showed moderate effects on vocabulary learning achievement (*g* = 0.62) and retention (*g* = 0.74), and a small effect on student attitude (*g* = 0.37). The adoption of the fixed effects model for attitude was due to homogeneous distribution, while the random effects model was used for achievement and retention because of heterogeneous distribution.

**Conclusion:**

Quizlet enhances vocabulary learning achievement, retention, and has small positive effect on learner attitude. Its integration into language education curricula is recommended to leverage these benefits. Further research is encouraged to explore the optimization of Quizlet and similar platforms for educational success.

## 1 Introduction

The exponential expansion of digital technologies within the realm of pedagogy has sparked an escalating curiosity in scrutinizing their effects on academic achievement among students. This surge in interest calls for a thorough examination of how these technological tools are reshaping educational practices and outcomes. Students are frequently referred to as “Digital Natives” on account of their innate fluency with various technological devices such as computers, the internet, and video games (Prensky, 2009). This inherent proficiency has been pivotal in driving the integration of digital tools in educational settings. The seamless incorporation of these technologies into classrooms, particularly language learning classrooms, highlights the evolving dynamics of modern education and emphasizes the need for empirical research to assess their impact. The field of language education has undergone a significant transformation due to the increasing influence of technology, resulting in a shift towards the integration of computers, mobile devices, and technology into teaching and learning practices (Aprilani, 2021). This paradigm shift demonstrates the critical role of technology in facilitating innovative teaching and learning strategies and thus improving the quality and accessibility of education. This integration has not only reshaped traditional educational methodologies but has also necessitated the incorporation of information technology (IT) into the teaching and learning process (Eady and Lockyer, 2013). As a result, the academic community is increasingly focused on understanding the impact of these changes on pedagogical practices, teaching and learning processes and student outcomes. In the domain of language education, the utilization of mobile technology has the capacity to transcend the constraints imposed by conventional learning methodologies in terms of spatial and temporal limitations, which ultimately caters to the individualized learning requirements of contemporary tertiary level scholars (Lin and Chen, 2022). This situation emphasizes the importance of investigating the effectiveness of mobile technologies in improving the quality of the learning process in language education and meeting the different needs of learners. Moreover, the global application of technology in English teaching and learning has been instrumental, benefiting both teachers and learners by facilitating classroom activities and accelerating language acquisition through the use of technology and its services (Nguyen and Van Le, 2022). Moreover, the integration of technology within the language learning milieu cultivates a heightened sense of self-directed and malleable learning methodology. Studies conducted on the domain of Computer-Assisted Language Learning (CALL) and Mobile-Assisted Language Learning (MALL) have inferred that the application of technological tools in the language learning process especially in the acquisition of vocabulary, particularly for non-native speakers, can be an efficacious strategy (McLean et al., 2013; Chatterjee, 2022). This body of research provides a compelling rationale for investigating specific digital tools like Quizlet and their potential to enhance language learning. As this digital transformation in language education continues, it becomes critical to examine specific digital tools and their unique contributions to this evolving educational paradigm, especially how they combine traditional methods with innovative technology-based strategies. Amidst this technological revolution in education, the role of specific tools such as Quizlet becomes increasingly significant. By focusing on Quizlet, this study aims to bridge the gap in the literature regarding the effectiveness of digital tools in enhancing vocabulary acquisition among language learners. Integrating technology into the language learning environment fosters a sense of independent and flexible learning methodology, especially in terms of vocabulary acquisition for non-native speakers. It is precisely at this point that the functionality and applicability of tools such as Quizlet, as part of the trend in digital education, becomes important in representing an intersection between traditional learning methodologies and modern, technology-enhanced approaches. Therefore, this study seeks to contribute to the broader discourse on digital education by examining the impact of Quizlet on vocabulary learning, retention and attitude, thereby offering insights into its role as a transformative tool in language education. By systematically reviewing and meta-analyzing existing literature, our study aims to provide a definitive assessment of Quizlet’s role in the digital education landscape, highlighting its potential as a transformative educational tool.

## 2 Literature review

In light of this reality, a myriad of applications with a focus on improving cognitive and emotional aspects of learners have surfaced on the internet, a substantial proportion of which can be readily downloaded and employed by users without incurring any costs. This proliferation of digital resources presents a convenient and easily accessible means for language learners to supplement their vocabulary acquisition, retention, motivation and attitude endeavors. An exemplar of such innovative technological solutions is Quizlet, a widely utilized online platform that provides a diverse array of educational tools, comprising interactive flashcards, gamified activities, and evaluation assessments, among others.

Andrew Sutherland designed a learning aid in 2005 that facilitated his academic excellence in French vocabulary assessment. He imparted it to his peers, and it resulted in a similar achievement in their respective assessments. Quizlet has since emerged as a powerful educational resource that has gained immense popularity, serving more than 60 million students and learners each month. Its widespread usage spans a wide range of disciplines, including mathematics, medicine, and foreign language acquisition, among others (Quizlet, 2023). Quizlet provides a platform that enables learners to curate personalized study materials consisting of conceptual units coupled with their corresponding definitions or elucidations. Learners engage with these instructional modules through varied modes of learning, such as flashcards, games, and quizzes (Fursenko et al., 2021). It is a popular web-based platform that offers a range of study tools, including flashcards, games, and quizzes. Quizlet is a well-known online learning application that enables users to build and study interactive resources like games and flashcards. According to Quizlet (2023), learning can be improved by using it in a variety of contexts and areas. The Quizlet mobile application is particularly effective for constructing vocabulary content. It has been proposed as a convenient and pleasurable method for acquiring vocabulary knowledge (Davie and Hilber, 2015). Within the app, users can access vocabulary “sets” created by other users, or they can generate their own sets and access them as flashcards or through a gaming interface (Senior, 2022). Quizlet is renowned for its distinctive attributes that pertain to the creation of flashcards, multilingual capacity, and the ability to incorporate images, among other forms of diverse exercises. However, it lacks the provision of scheduling and expanded retrieval intervals as the learning process advances. There are various ways in which vocabulary sets in Quizlet can be disseminated, including but not limited to printing, embedding, incorporating URL links, and utilizing QR codes. These options provide a range of alternatives for learners to study at their preferred pace, allowing for individualized and autonomous learning experiences (Waluyo and Bucol, 2021). This shift places Quizlet within a broader movement towards digitalization in education, juxtaposing its role with other emerging educational technologies.

The implementation of Quizlet in language education can potentially augment the learning experience and facilitate the assimilation of new linguistic concepts in a more efficient and engaging manner (Wang et al., 2021). While many educators and students have claimed that Quizlet improves cognitive and emotional learning outcomes, the empirical evidence supporting this claim has been mixed. Alastuey and Nemeth (2020) discussed the effects of Quizlet on vocabulary acquisition, highlighting the cognitive, affective, and motivational benefits for students creating their own learning material. However, Nguyen and Van Le (2022) pointed out the limited empirical research on the effectiveness of Quizlet, indicating a gap in the evidence. İnci (2020) reported that the use of the application in foreign language lessons improved learner engagement of learners. On the other hand, Berliani and Katemba (2021) found that most students considered Quizlet effective in learning vocabulary, supporting the positive impact on cognitive and emotional learning outcomes. Additionally, Setiawan and Wiedarti (2020) reported that the Quizlet application positively influences students’ performance and motivation in learning vocabulary. Therefore, while some studies support the claim of Quizlet’s positive effects on cognitive and emotional learning outcomes, there is also a need for further empirical research to provide a more comprehensive understanding of its impact. Nguyen et al. (2022) found that Quizlet positively influences students’ performance and autonomy in learning vocabulary (Nguyen et al., 2022). Similarly, Sanosi (2018) conducted an experimental-design study that investigated the effect of Quizlet on vocabulary acquisition, highlighting its potential for enhancing vocabulary learning (Anjaniputra and Salsabila, 2018; Sanosi, 2018) reported that Quizlet fostered learners’ engagement and persistence in vocabulary learning, indicating its usefulness as a learning tool (Anjaniputra and Salsabila, 2018). Furthermore, Alastuey and Nemeth (2020) examined the use of Quizlet in an urban high school language arts class and demonstrated that students using Quizlet outperformed those in the Non-Quizlet group on weekly vocabulary tests, emphasizing its positive impact on vocabulary acquisition (Alastuey and Nemeth, 2020; Setiawan and Wiedarti, 2020) found that the Quizlet application positively influenced students’ performance and autonomy in learning vocabulary, further supporting its effectiveness (Setiawan and Wiedarti, 2020). These findings align with the research by Körlü and Mede (2018), which indicated that Quizlet had a positive impact on students’ performance and autonomy in vocabulary learning (Körlü and Mede, 2018). Also, several studies have demonstrated the positive influence of Quizlet on student’s cognitive learning such as vocabulary learning and retention (Barr, 2016; Özer and Koçoğlu, 2017; Ashcroft et al., 2018; Körlü and Mede, 2018; Sanosi, 2018; Andarab, 2019; Çinar and Ari, 2019; Toy, 2019; Arslan, 2020; Chaikovska and Zbaravska, 2020; Tanjung, 2020; Van et al., 2020; Akhshik, 2021; Aksel, 2021; Fursenko et al., 2021; Ho and Kawaguchi, 2021; Kurtoğlu, 2021; Setiawan and Putro, 2021; Atalan, 2022; Lin and Chen, 2022; Nguyen and Van Le, 2022, 2023).

However, these studies also show that the effectiveness of tools such as Quizlet can vary considerably depending on various factors such as students’ readiness, the teaching-learning process, the learning environment and the type of language skill targeted. Along these lines, a critical review of how Quizlet affects learning outcomes is still evolving. While some studies demonstrate the positive effects of technologies such as Quizlet, others offer a more cautious view, painting the other side of the coin and pointing to limitations and variable outcomes in different educational contexts. For example, some students prefer Quizlet because of its convenience, usefulness, practicality and effectiveness, while others express dissatisfaction with certain features and errors (Pham, 2022). Moreover, the effectiveness of Quizlet in vocabulary learning has been a subject of research, with some studies indicating its success in enhancing vocabulary acquisition and retention (Körlü and Mede, 2018; Al-Malki, 2020; Mykytka, 2023), while others suggest that its use does not necessarily lead to autonomous learning (Setiawan and Wiedarti, 2020).

Given the varied and sometimes contradictory findings of related studies in the literature, a more general, systematic and comprehensive approach is needed to understand the real impact of Quizlet on learning outcomes. While generally positive in the literature, the different perspectives and diverse results reported highlight the complexity of evaluating the effectiveness of digital learning tools such as Quizlet. This underlines the need for a more comprehensive, nuanced and evidence-based evaluation and is the main focus of the current research. Unfortunately, there is little and contradictory empirical data to support Quizlet’s impact on learning outcomes. As a result, the purpose of this research is to consolidate and assess the body of knowledge on the effect of Quizlet on learning outcomes through a thorough meta-analysis and systematic review. This research aims to fill the existing gap in empirical research, provide valuable insights, and contribute to the literature in terms of having a final say on the broader understanding of Quizlet’s effectiveness in language learning. Consequently, a comprehensive and systematic review of the existing literature is warranted to evaluate the impact of Quizlet especially on student emotional learning outcomes. This meta-analysis aims to fill this gap by synthesizing the available research on Quizlet and providing a quantitative assessment of its effectiveness in enhancing student cognitive and emotional learning outcomes. By conducting a systematic review and comprehensive meta-analysis, this research aims to determine whether Quizlet’s utilization leads to a significant difference in student outcomes in these key areas compared to traditional or alternative learning methods. Through a rigorous and transparent synthesis of the empirical evidence, this study seeks to shed light on the potential of Quizlet to improve student cognitive and emotional learning outcomes and inform future research and practice in the field of digital education.

The detailed literature review revealed a scarcity of quantitative studies suitable for a meta-analysis, particularly concerning the effects of Quizlet on aspects such as student motivation, confidence, learner engagement, and anxiety. Consequently, this study focuses on quantifying the impact of Quizlet on foreign language learners’ vocabulary learning achievement, retention, and attitude through a systematic review and comprehensive meta-analysis. The study investigates whether the use of Quizlet results in a significant difference in student scores in these areas compared to other learning methods.

## 3 Methodology

### 3.1 Model of the research

In this study, we conducted a comprehensive examination of quantitative research focusing on the application of Quizlet in vocabulary learning. The selection of studies for this meta-analysis was guided by systematic and rigorous methods, as recommended by the PRISMA guidelines. In accordance with PRISMA guidelines, researchers have employed systematic review and meta-analysis methodologies to ensure transparency, reproducibility, and rigor in their studies (Page et al., 2021). The PRISMA guidelines provide a comprehensive checklist for reporting systematic reviews and meta-analyses, encompassing various aspects such as study selection, data extraction, and synthesis methods (Page et al., 2021). Adhering to these guidelines enhances the quality and reliability of the research findings, thereby contributing to evidence-based decision-making in diverse fields.

This study involved a comprehensive search of literature from the inception of Quizlet in 2005 to the present year, 2023. Our focus on the period from 2016 to 2023 is based on the emergence of a pivotal study in 2016, which was the first to explore the impact of Quizlet on the identified outcomes. Our analysis encompassed a broad spectrum of educational settings and demographics, reflecting the diverse populations engaged in using this tool. The core intervention we scrutinized was Quizlet’s utilization for enhancing vocabulary learning, its retention, and its influence on learner attitudes. To gauge Quizlet’s efficacy, we included studies that provided a comparative analysis between Quizlet and traditional learning methodologies or other educational technologies. This comparative approach enabled us to assess the relative effectiveness of Quizlet in achieving the desired educational outcomes. Our primary outcomes of interest were learners’ achievement in vocabulary acquisition, the retention of this knowledge over time, and their attitudes towards the use of Quizlet as a learning tool. The temporal scope of our review was strategically chosen, with 2016 marking the emergence of a pivotal study that set a precedent in this research area, thereby shaping the subsequent investigations into Quizlet’s impact in educational contexts. In accordance with PRISMA guidelines, we have employed a meta-analytic survey approach to determine the effect size of Quizlet’s impact on foreign language learners’ vocabulary learning, retention, and attitudes. This includes a thorough evaluation of study quality, risk of bias, and the applicability of findings.

### 3.2 Data collection and coding

**Figure 1:**
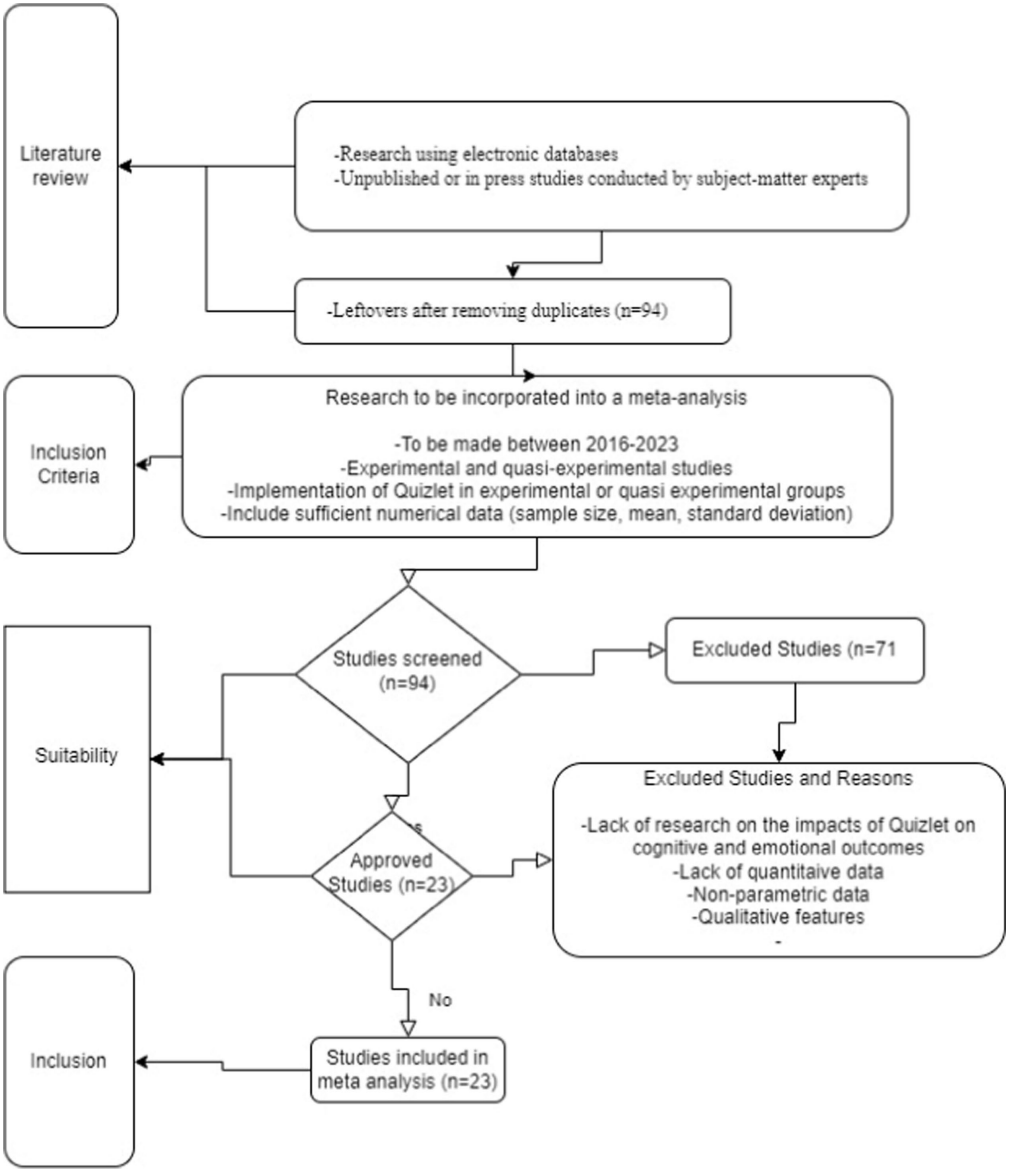
Flow chart showing the process of scanning the literature and inclusion of accessed studies in meta-analysis.

In the course of gathering data for this research, an examination of diverse databases was conducted, encompassing the YÖK national thesis center, Google Scholar, Selcuk University Academic Search Engine, DergiPark, Proquest, Sage Journals, Eric, Wiley Online Library, Taylor & Francis Online, Science Direct, Jstor, and Springer Link databases. Throughout this phase, the fundamental concepts under consideration were “Quizlet” and “mobile flashcards.” In the preliminary scrutiny of this research, a comprehensive total of 94 studies were discerned. Nevertheless, 71 of these studies were omitted from the meta-analysis owing to their qualitative nature, irrelevant outcomes such as absence of exploration into academic achievement, learning retention, student attitude, and the format such as the transformation from thesis to article format. A significant number of studies were based on qualitative research methodologies. While these studies provide valuable insights, our meta-analysis focused on quantifiable outcomes that could be statistically analyzed. Therefore, studies that primarily employed qualitative methods such as interviews, narrative analysis, or case studies were excluded. Several studies did not align with the specific outcomes of interest for our research. Our meta-analysis aimed to explore the impact of “Quizlet” on academic achievement, learning retention, and student attitudes. Studies that did not investigate these specific outcomes, or that focused on peripheral aspects not directly related to our research questions, were omitted. This included studies that might have used similar tools or technologies but did not measure the outcomes relevant to our analysis. For studies that were available in both thesis and article formats, we chose to exclude the thesis versions. This decision was made because our research aimed to understand how the condensation and refinement involved in transforming a thesis into a journal article could impact the presentation and interpretation of research findings. Including both formats of the same study could lead to redundancy and skew the meta-analysis results. Flow chart in (Figure 1) shows the process of scanning the literature and inclusion–exclusion of accessed studies in meta-analysis.

To ensure systematic data analysis, we employed a two-stage coding process. In the first stage, each study was preliminarily coded based on its relevance to our key concepts. This step helped in filtering out studies that did not directly contribute to our research questions. The second stage involved a more detailed coding procedure, where two independent researchers coded the remaining studies for more specific variables such as research methodology, population, outcomes measured, and main findings. Any discrepancies between the coders were resolved through discussion and consensus, ensuring a high degree of inter-coder reliability. This step was crucial in maintaining the objectivity and consistency of the data coding process. The validity and reliability calculations related to the coding process are discussed in detail in the section titled “Reliability and validity of the research” of this study.

### 3.3 Inclusion criteria

Prescribed protocols are advised for the execution of meta-analyses, as delineated by Field and Gillett (2010) and Bernard et al. (2014). Fundamentally, these scholars advocate for a meticulous assessment of the literature search process and a thorough evaluation of the selected studies with regard to potential publication bias, which should be undertaken prior to the initiation of pertinent statistical analyses. In order to be incorporated into the meta-analysis, prospective studies were required to conform to several specific eligibility criteria. Firstly, an eligible study had to investigate the impact of Quizlet. Secondly, the study had to make a comparative assessment of the impact of Quizlet in relation to a control group. Prospective-pre-post designs that did not incorporate a control or comparison group were excluded from consideration, as they failed to account for potential influences stemming from natural development or extraneous variables. Additionally, in order to meet the inclusion criteria, a study had to be conducted within an educational setting or possess a clear relevance to educational outcomes. This encompassed all levels of education, spanning from tertiary to secondary and primary levels. Furthermore, an eligible study was required to furnish adequate data for the computation of an effect size. Figure 1 reflects flow chart of the process of scanning the literature and inclusion of accessed studies in meta-analysis.

In order to ensure comprehensive coverage, a meticulous internet search was carried out according to the inclusion criteria mentioned in Figure 1 and a total of 94 studies were initially identified related to the topic. During the screening process, a significant portion of these studies were excluded due to several factors. Excluded studies included non-parametric data, qualitative features, quantitative data, and studies that did not examine the effects of Quizlet on cognitive and emotional outcomes. Exclusion of studies with qualitative characteristics was necessary since they did not comply with the quantitative methodology needed for this meta-analysis. Some studies were excluded due to the lack of control groups. The inclusion of both control and experimental groups in studies is crucial for ensuring the validity of research findings. The importance of experimental and quasi-experimental designs for generalized causal inference in meta-analyses of research is emphasized in literature (Preece, 1983; Anderson-Cook, 2005; Morris, 2007). By meticulously applying these inclusion criteria, the meta-analysis carefully selected studies with precision. This thorough approach ensured that only the most relevant and suitable sources were included, thereby enhancing the overall quality and reliability of the analysis. To measure the effects of the Quizlet on vocabulary learning achievement, retention, and attitude, 23 carefully selected studies that met the inclusion requirements made up the study’s sample. These studies had a range of sample sizes and covered a range of study types. Table 1 provides a detailed summary of the publication year, study type, research courses, and sample sizes of the included studies, giving a clear picture of the make-up of the meta-analysis sample.

**Table 1:** Features of the studies included in the meta-analysis.

Characteristic	Publication year of research	*n*	Type of research	*n*	Courses of research	*n*	The level of education at the school	*n*	The place of the study	*n*
1	2023	1	Article	15	English Language	22	University	14	Studies in Turkey	9
*2*	2022	3	Dissertations (MA)	5	Chinese Language	1	Elementary	5	Studies in Ukraine	2
3	2021	7	Conference Proceedings	3			High	4	Studies in Vietnam	4
4	2020	4							Studies in the USA	1
5	2019	3							Studies in Saudi Arabia	1
6	2018	3							Studies in Indonesia	2
7	2017	1							Studies in Japan	2
8	2016	1							Studies in Iran	1
9									Studies in China	1
Total		23		23		23				23

### 3.4 Reliability and validity of the research

When performing meta-analysis studies, it is imperative to methodically assemble descriptive data that highlights the important aspects of the included research. Careful data collection is necessary for this, where relevant information is meticulously recorded to facilitate additional analysis. The process of coding in meta-analysis studies is crucial for converting descriptive data into numerical data, enabling statistical analysis (Neyeloff et al., 2012). Coding processes are essential for the synthesis of data and are used to convert descriptive data into a format that can be subjected to statistical analysis (Berkeljon and Baldwin, 2009). According to Karadağ et al. (2015), coding is a methodical and scientific approach that is utilized to extract pertinent and accurate data from the massive amount of data that is collected throughout the investigations.

In the coding process of this study to guarantee the accuracy of the coding process, a stringent methodology was developed. The coding was done in compliance with the coding form, which was established prior to the analysis. Creating a unique coding system that was both general and distinct enough to capture the features of any kind of research was the primary goal of this process. The coding process was done independently by two experts, each with extensive knowledge and expertise in the field. The coding forms completed by the first and second experts were carefully compared in order to assess the level of agreement between them. The calculation of Inter-rater Reliability (IRR) can be quantitatively assessed using the formula agreement/(agreement + disagreement) x 100. This formula enables a quantitative assessment of the consistency between the two experts’ coding (Hripcsak and Rothschild, 2005). It is important to note that the calculation of IRR is crucial in various fields, including medical informatics, forensic psychology, and educational measurement (Hripcsak and Heitjan, 2002; Cook et al., 2008; Guarnera and Murrie, 2017). The use of this formula allows for the measurement of specific agreement, which is essential in quantifying interrater reliability and assessing the reliability of a gold standard in various studies (Hripcsak and Heitjan, 2002). The calculated Inter-rater Reliability (IRR) score provided a measure of the degree of agreement between the two experts, indicating the reliability of the coding process. In this study, the reliability was determined to be 98%, signifying a high level of concordance between the experts’ assessments. The high reliability score of this meta-analysis enhances the overall reliability and robustness of the results by providing assurance about the coding process’s precision and consistency.

### 3.5 Data analysis procedure

This study utilizes Hedges’s *g* as the measurement unit for effect size. A significance level of 95% is established. The total effect size is then determined by first calculating the effect sizes for each study included in the meta-analysis. Two models, namely fixed effects and random effects are employed to determine the overall effect size.

The effect sizes obtained from the analyses were interpreted using the effect size classification proposed by Thalheimer and Cook (2002) and Hunter and Schmidt (2015). These researchers discuss methods of meta-analysis and provide insights into the interpretation of effect sizes. According to them, the classification of effect sizes as small (0.15 ≤ Hedges’s *g* < 0.40), moderate (0.40 ≤ Hedges’s *g* < 0.75), large (0.75 ≤ Hedges’s *g* < 1.10), very large (1.10 ≤ Hedges’s *g* < 1.45), and excellent (Hedges’s *g* ≥ 1.45). These references provide a comprehensive understanding of the interpretation of effect sizes, aligning with the specified ranges for Hedges’s *g*.

## 4 Findings

This study’s data analysis produced several noteworthy conclusions, which are discussed in more detail below.

### 4.1 Effect size

The effect size, represented as “d,” was determined as the result of dividing the discrepancy between the treatment conditions by the amalgamated standard deviation of the two study groups (Borenstein et al., 2021). Cohen’s *d* or Hedges’ *g* represent the effect size when utilizing contrast groups (Hedges, 1983; Hedges and Olkin, 1985; Cohen, 1988; Hartung et al., 2008; Borenstein et al., 2009). Hedges’ *g* is particularly useful for small sample sizes and is preferred when the studies being compared have different sample sizes or variances (Light et al., 1994).

The decision on whether to use a fixed effects model or a random effects model for calculating effect sizes in a study is crucial. Calculating effect sizes “d” and “g” by dividing the discrepancy between the means of each group’s experimental and control cohorts by their pooled standard deviations is a purposeful and meaningful procedure (Borenstein et al., 2009). While the random effects model aims to generalize findings beyond the included studies by assuming that the selected studies are random samples from a larger population, the fixed effects model is ideal for drawing conclusions on the studies included in the meta-analysis (Cheung et al., 2012). The random effects model is a good option for meta-analysis when the goal is to make the results more broadly applicable than the individual research. A random effects model takes into account both within- and between-study variation, making it more cautious and producing a broader confidence interval (Ma et al., 2015). The second criterion is dependent upon the number of studies that are included in the meta-analysis. The fixed effects model is considered appropriate when the number of studies is less than five (Aydin et al., 2020). Studies are deemed homogenous if the variance in effect sizes amongst them is only attributable to sampling error; in a meta-analysis, this source of variation can be accounted for by employing the fixed effect model (Idris and Saidin, 2010). The random effects model assumes a normal distribution of genuine effect sizes and estimates the mean and variance of this distribution, whereas the fixed effects model assumes a common true effect size across all studies and calculates this common effect size (Spineli and Pandis, 2020). The third criterion is whether statistical heterogeneity exists between effect sizes. The random effects model must be used when heterogeneity is found, as explained by Tufanaru et al. (2015). A random effects model, which takes into account both within- and between-study variance, can be more suited if there is significant heterogeneity among the studies. Conversely, a fixed effects approach would be more appropriate if the trials are quite homogeneous (Danos, 2020). It is important to consider the assumptions and implications of each model when making this decision, as the choice of model can impact the interpretation and generalization of the results (Konstantopoulos, 2006).

In this study, the statistical data on vocabulary learning and retention of Quizlet application were interpreted according to the random effects model since they showed heterogeneous distribution (see Tables 2, 3), and the data on student attitude were interpreted according to the fixed effects model since they showed homogeneous distribution (see Table 4).

**Table 2:** Homogeneity test results: *Q*-statistic, *I*^2^ and tau-square statistics assessing the impact of Quizlet on vocabulary learning achievement.

*Q* value	df (Q)	*p*-value	*I*^2^value	τ^2^
113.069	20	0.000	82.312	0.284

**Table 3:** Homogeneity test results: *Q*-statistic, *I*^2^ and tau-square statistics assessing the impact of Quizlet on vocabulary retention.

*Q* value	df (*Q*)	*p*-value	*I*^2^value	τ^2^
**20.997**	**4**	**0.000**	**80.947**	**0.259**

**Table 4:** Homogeneity test results: Q-statistic, *I*^2^ and tau-square statistics assessing the impact of Quizlet on student attitude.

*Q* value	df (*Q*)	*p*-value	*I*^2^value	τ^2^
2.003	1	0.157	50.081	0.065

### 4.2 The meta-analysis outcomes pertaining to the influence of Quizlet on vocabulary learning achievement

In pursuit of addressing the primary research query, the study sought to ascertain the extent to which Quizlet, as supported by experimental study findings, contributes to students’ vocabulary learning. To unravel this quandary, meticulous analyses were conducted on the pertinent data extracted from the studies encompassed within the research. Heterogeneity in the context of meta-analysis refers to sampling error and the variation in results seen across many research papers (Borenstein et al., 2009). To evaluate the degree to which the conclusions of each research study are influenced by both the sampling error and the fluctuation or population variance in the estimated effect size, it becomes essential to conduct a heterogeneity assessment. The results of the heterogeneity test also help to identify if the study fits better with a fixed effect model or a random effect model. As a result, any of these impact models is used to calculate the effect magnitude or summarizing effect of the study’s findings for further research. In this work, heterogeneity testing is examined using Q-statistics in conjunction with its *p*-value, *I*^2^ and τ^2^ parameters, all of which are listed in Table 2.

According to the homogeneity test in Table 2, the average effect size Q-statistical value of the Quizlet on vocabulary learning is calculated as 113.069 at 20 degrees of freedom at 95% significance level and is found to be statistically significant (*Q* = 113.069; *p* < 0.05). According to the *Q*-value results of the research data, it can be said that the distribution is heterogenous. The tau-square value (τ^2^), which estimates the variance of the true mean effect size, is calculated as (τ^2^) 0.284, and the *I*^2^ statistic is calculated as 82.312. This *I*^2^ value calculated for the vocabulary learning variable indicates that we can explain 82.312% of the variance in the average effect size calculated in the studies included in the meta-analysis with the data we have and indicates a high level of heterogeneity.

**Figure 2:**
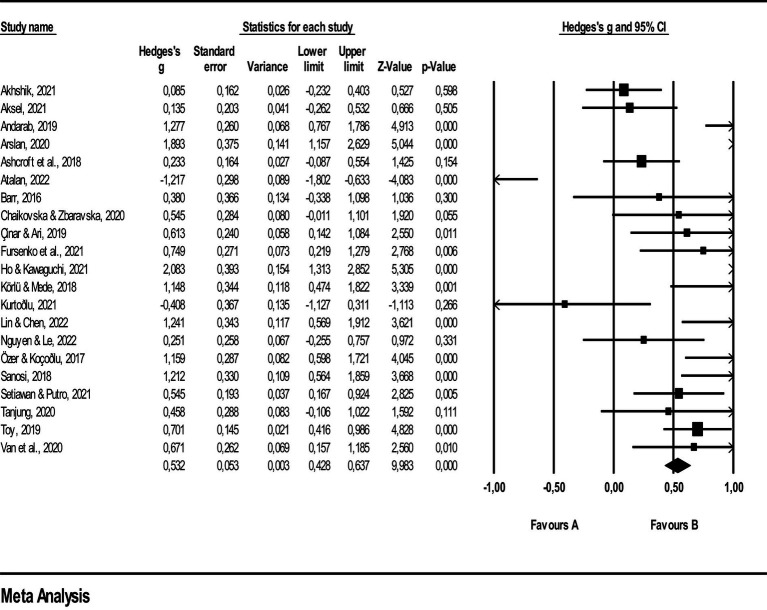
Effect size values related to vocabulary learning achievement.

In Figure 2, the lines flanking the squares represent the lower and upper bounds of effect sizes within a 95% confidence interval, while the rhombus indicates the overall effect size of the studies. Upon examination, the smallest effect size is −0.408, and the largest is 2.083. The weight percentage provided alongside the effect size values quantitatively represents the contribution of each study to the overall outcome of the meta-analysis.

The results that are provided in Table 5 indicate that Quizlet has moderate impact on vocabulary learning. These empirical results highlight the critical need and effectiveness of using Quizlet as an instructional tool to help students develop higher order lexical knowledge. Using the Classic Fail-Safe N analysis, a technique used to determine the strength of the meta-analysis under consideration, it was confirmed that Quizlet has a modest effect (effect size, *g* = 0.62) on the improvement of vocabulary proficiency. Table 6 presents the Classic Fail-Safe N Analysis of this examination, providing additional insight into the validity and strength of the determined outcomes. Classic fail-safe N analysis is utilized to determine the stability of results and to identify the potential impact of unpublished studies on the overall conclusions of a meta-analysis (Erford et al., 2010) and it provides an indication of the robustness of the findings and is employed to evaluate the stability of the meta-analytic results (Acar et al., 2017).

**Table 5:** Average effect sizes and confidence interval lower and upper values by effect model.

Model	*N*	Hedges’s *g*	%95 confidence interval		Heterogeneity		
			Lower limit	Upper limit	*Q*-value	*P*	*I* ^2^
Fixed effect model	21	0.532	0.428	0.637	113.069	0.000	82.312
Random effect model	21	0.628	0.370	0.885			

**Table 6:** Classic fail-safe N analysis.

The strength of meta-analysis	
*Z* value for observed studies	10.56395
*P*-value for observed studies	0.00000
Alpha	0.05000
Tails	2.00000
*Z* for alpha	1.95996
Number of observed studies	21.00000
Number of missing studies that would bring *p*-value to >alpha	590.00000

Based on the Classic Fail-Safe N analysis, it becomes apparent that an additional 590 studies would be necessary to potentially alter the conclusion drawn from the meta-analysis, suggesting that Quizlet’s impact on vocabulary learning is either negligible or negative (*p* < 0.05). The inclusion of these 590 studies reporting no substantial impact of Quizlet on vocabulary learning would be pivotal to reconsidering the overall outcome of the meta-analysis. Their incorporation would significantly enhance the breadth of evaluation regarding Quizlet’s relationship with vocabulary learning, enriching our understanding of its potential effects as an instructional tool.

**Figure 3:**
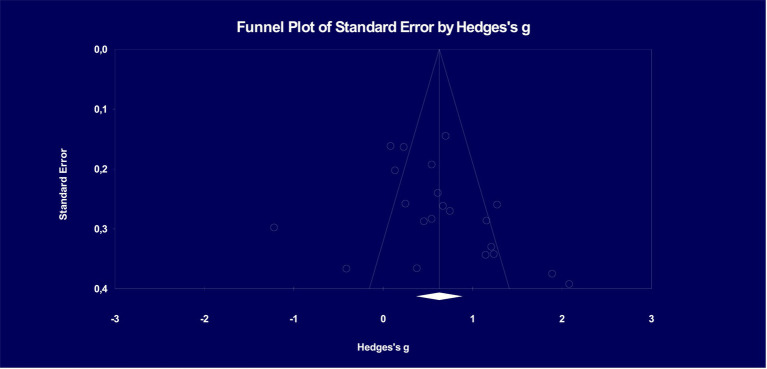
Funnel plot on publication bias of studies examining the effect of Quizlet on vocabulary learning achievement.

Figure 3 illustrates the distribution of effect sizes in accordance with Hedges’s funnel chart (Funnel plot of precision). The funnel plot is a widely used tool in meta-analysis to visually assess the presence of publication bias and small-study effects (Egger et al., 1997). It provides a graphical display of the relationship between the effect size estimates and a measure of study precision, such as the standard error or sample size (Kiran et al., 2016). Funnel plots are particularly useful in exploring sources of heterogeneity and bias in meta-analyses (Schild and Voracek, 2013). These graphical representations not only aid in visualizing the effect size distribution but also underscore the importance of assessing publication bias in meta-analytical studies, ensuring a comprehensive and unbiased evaluation of Quizlet’s impact on vocabulary learning achievement.

According to Figure 3, it becomes evident that the investigations fail to exhibit an asymmetrical distribution with respect to the overall effect size. The funnel’s edge in the graphic is marked by a ± slope. Figure 3 reveals that significant differences or anomalies in distribution are conspicuously missing. To put it differently, the distribution does not display a pronounced concentration on one side. The graphic clearly conveys that there are many studies that are located outside of the funnel, highlighting the significant diversity that exists within this cohort and making it possible to say that the group is heterogeneous. Our thorough investigation turned up no concrete proof of publication bias among the variety of research we included in our meta-analysis. The absence of an asymmetric clustering at a singular point within the distribution signifies that the study sample does not exhibit a predisposition towards favoring the Quizlet, thereby enhancing the reliability of this meta-analysis study.

Table 7 meticulously catalogues the results of the Begg and Mazumdar rank correlation test. This statistical test evaluates the relationship between the standardized treatment effect and the variance of the treatment effect using Kendall’s tau (Gjerdevik and Heuch, 2014).

**Table 7:** Begg and Mazumdar rank correlation.

Kendall’ *S* Statistic (P-Q)	54.00000
**Kendall’s tau without continuity correction**	
Tau	0.25714
*Z* value for tau	1.63063
*p*-value (one-tailed)	0.05148
*p*-value (two-tailed)	0.10297
**Kendall’s tau with continuity correction**	
Tau	0.25238
*Z* value for tau	1.60044
*p*-value (one-tailed)	0.05475
*P*-value (two-tailed)	0.10950

In Table 7, the Begg and Mazumdar rank correlation test has unveiled that the composite study sample integrated into the meta-analysis does not demonstrate any signs of bias (tau *b* = 0.25; *p* > 0.05). Consequently, the findings derived from the scrutiny of effect sizes originating from the constituent studies are deemed to possess a high degree of reliability. This signifies that the inferences drawn from the meta-analysis concerning the influence of the Quizlet on the assessed parameters can be characterized as sturdy and trustworthy. These results highlight the validity and reliability of the conclusions drawn from our research, which can be regarded as robust and unwavering.

### 4.3 The meta-analysis outcomes pertaining to the influence of Quizlet on vocabulary retention

The secondary inquiry in this study aimed to ascertain the impact of Quizlet on retaining vocabulary. To address this, a meticulous analysis of relevant data gleaned from the research was undertaken. Homogeneity assessments, as detailed in Table 3, were conducted to ascertain the suitability of employing either the fixed effects model or the random effects model for computing the effect sizes associated with the influence of Quizlet on vocabulary retention. These assessments aimed to discern the best approach to quantify the impact of Quizlet on the retention of vocabulary across different study conditions or populations. Moving from these methodological decisions, the subsequent focus lay in interpreting the implications of Quizlet’s influence on vocabulary retention within diverse study contexts.

As a result of the homogeneity test, the average effect size *Q*-statistical value of the Quizlet on vocabulary retention is calculated as 20.997 at 4 degrees of freedom at 95% significance level and is found to be statistically significant (*Q* = 20.997; *p* < 0.05). According to the Q-value results of the research data, it can be said that the distribution is heterogenous. The tau-square value (τ^2^), which estimates the variance of the true mean effect size, is calculated as (τ^2^) 0.259, and the *I*^2^ statistic is calculated as 80.947. This *I*^2^ value calculated for the vocabulary retention variable indicates that we can explain 80.947% of the variance in the average effect size calculated in the studies included in the meta-analysis with the data we have and indicates a high level of heterogeneity.

**Figure 4:**
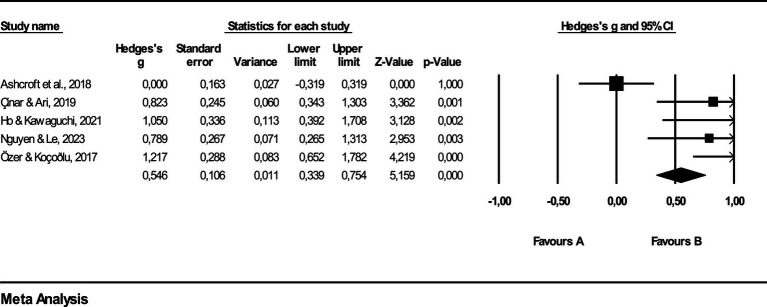
Effect size values related to vocabulary retention.

In Figure 4, the lines bordering the squares depict the range of effect sizes encompassed by a 95% confidence interval, with the rhombus denoting the aggregate effect size derived from the studies. Analysis reveals effect sizes ranging from 0.000 to 1.217. Additionally, the weight percentage accompanying each effect size quantifies the relative impact of individual studies on the collective result of the meta-analysis. This visual representation not only delineates the variability in effect sizes but also emphasizes the influence of each study on the overall outcome, providing a nuanced understanding of the meta-analytical findings.

The findings outlined in Table 8 demonstrate that Quizlet exerts a moderate impact on vocabulary retention (Hedges’ *g* = 0.743). Employing the Classic Fail-Safe N analysis, a method aimed at gauging the robustness of the meta-analysis, affirmed the moderate effect of Quizlet (*g* = 0.74) in advancing vocabulary retention. Table 9 within the study further expounds upon this analysis, offering deeper insights into the credibility and potency of the conclusions drawn from the investigation. This robust analysis not only validates the efficacy of Quizlet but also emphasizes its substantive contribution to enhancing vocabulary retention among learners.

**Table 8:** Average effect sizes and confidence interval lower and upper values by effect model.

Model	*N*	Hedges’s *g*	%95 confidence interval		Heterogeneity		
			Lower limit	Upper limit	*Q*-value	*P*	*I* ^2^
Fixed effect model	5	0.546	0.339	0.754	20.994	0000	80.947
Random effect model	5	0.743	0.241	1.245			

**Table 9:** Classic fail-safe N analysis.

The strength of meta-analysis	
*Z* value for observed studies	6.11025
*P*-value for observed studies	0.00000
Alpha	0.05000
Tails	2.00000
*Z* for alpha	1.95996
Number of observed studies	5.00000
Number of missing studies that would bring *p*-value to >alpha	44.00000

**Figure 5:**
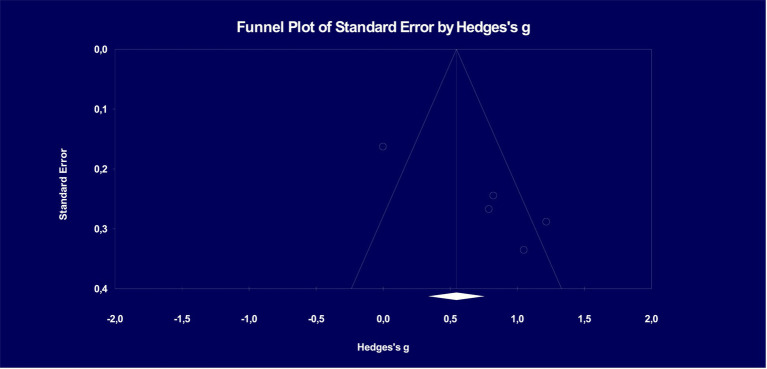
Funnel plot on publication bias of studies examining the effects of Quizlet on vocabulary retention.

Classic Fail-Safe N analysis reveals that an additional 44 studies would be required to potentially modify the conclusion drawn from the meta-analysis. This indicates a possibility that Quizlet’s impact on vocabulary retention might be insignificant or even negative (*p* < 0.05). The inclusion of these 44 studies, which report no substantial impact of Quizlet on vocabulary retention, would be crucial in reconsidering the overall outcome of the meta-analysis. Their inclusion would significantly broaden the scope of the evaluation concerning Quizlet’s association with vocabulary retention, thereby enriching our understanding of its potential as an instructional tool. Additionally, Figure 5 demonstrates the distribution of effect sizes based on Hedges’s funnel chart (Funnel plot of precision).

Figure 5 displays a funnel plot illustrating the distribution of effect sizes across studies. The funnel in the plot is bounded by a ± slope. The graphic indicates that some studies fall outside the slope curve, suggesting heterogeneity within the group.

Based on the data synthesized in Table 10, employing the Begg and Mazumdar rank correlation test revealed no indications of bias within the combined study sample used in the meta-analysis (tau *b* = 0.60; *p* > 0.05). As a result, the scrutiny of effect sizes extracted from these studies is considered highly reliable. This substantiates the robustness of the conclusions drawn from the meta-analysis, specifically regarding Quizlet’s influence on the evaluated parameters. These outcomes not only affirm the validity and reliability of our research findings but also underscore the steadfastness and solidity of the conclusions derived. It emphasizes the credibility of our study’s outcomes, reinforcing the confidence in the observed effects of Quizlet on the assessed parameters.

**Table 10:** Begg and Mazumdar rank correlation.

Kendall’ *S* Statistic (P-Q)	6.00000
**Kendall’s tau without continuity correction**	
Tau	0.60000
*Z* value for tau	1.46969
*P*-value (one-tailed)	0.07082
*P*-value (two-tailed)	0.14164
**Kendall’s tau with continuity correction**	
Tau	0.50000
*Z* value for tau	1.22474
*P*-value (one-tailed)	0.11034
*P*-value (two-tailed)	0.22067

### 4.4 The meta-analysis outcomes pertaining to the influence of Quizlet on student attitude

**Figure 6:**
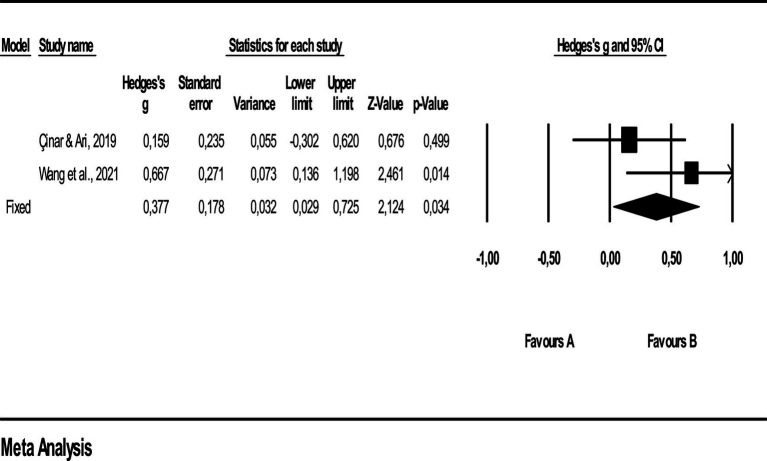
Effect size values related to student attitude.

As a result of the homogeneity test, the average effect size *Q*-statistical value of the Quizlet on student attitude is calculated as 2.003 at 1 degree of freedom at 95% significance level and is found to be statistically insignificant (*Q* = 2.003; *p* > 0.05). According to the *Q*-value results of the research data, it can be said that the distribution is homogenous. The tau-square value (τ^2^), which estimates the variance of the true mean effect size, was calculated as (τ^2^) 0.065, and the *I*^2^ statistic is calculated as 50.081. This *I*^2^ value calculated for the student attitude variable indicates that we can explain 50.081% of the variance in the average effect size calculated in the studies included in the meta-analysis with the data we have and indicates a high level of homogeneity. Since the results of the homogeneity test analysis and *I*^2^ statistics indicate that the studies on the average effect size of the Quizlet on student attitude does not differ statistically from each other, the analyses were calculated according to the fixed effects model (Figure 6).

It is seen that the weights of the studies in the meta-analysis are close to each other. It can be visually understood from the forest plot that the effect sizes are generally concentrated at a low level and the overall effect size is low in width.

The meta-analysis values of the results obtained from the 2 studies included in the meta-analysis are given in Table 11. Table 11 shows the homogeneous distribution value, average effect size and confidence intervals of the studies according to the effect model.

**Table 11:** Average effect sizes and confidence interval lower and upper values by effect model.

Model	*N*	Hedges’s *g*	%95 confidence interval		Heterogeneity		
			Lower limit	Upper limit	*Q*-value	*P*	*I* ^2^
Fixed effect model	2	0.377	0.029	0.725	2.003	0.157	50.081
Random effect model	2	0.395	−0.101	0.891			

The findings outlined in Table 11 demonstrate that Quizlet exerts a small impact on student attitude (Hedges’ *g* = 0.377). According to the fixed effects model, the lower limit of the 95% confidence interval is 0.029, the upper limit is 0.725. Classical Fail-Safe N analysis, Funnel Plot and Begg and Mazumdar Rank Correlation values could not be calculated since the number of studies examining the effect of Quizlet on student attitude according to the inclusion criteria of this study was limited to 2 in the literature.

## 5 Discussion

The objective of this meta-analysis was to analyze the overall results acquired from studies that examined the impact of Quizlet on foreign language learners’ cognitive and emotional outcomes such as vocabulary learning achievement, retention, and attitude. This study synthesized previous research to determine the Quizlet’s impact level. The trustworthiness of the research findings is demonstrated by the confidence intervals derived from the meta-small analysis. A thorough examination of the Quizlet’s effect on numerous outcome measures was made possible by the combination of experimental and quasi-experimental research.

To begin with, the study’s first research issue focuses on the impact of Quizlet on students’ vocabulary learning achievement as measured by experimental experiments. To address this topic, a meta-analysis incorporating 21 relevant studies was conducted. To determine whether the fixed effects model or the random effects model is appropriate for the research, a homogeneity test was first carried out. The homogeneity test results showed a statistically significant difference (*Q* = 113.069; *p* < 0.05), indicating a diverse distribution of effect sizes among the studies. A further indication of the significant degree of variability among the studies was the obtained *I*^2^ value of 82.312%. The random effects model was used to calculate effect estimates because of the significant heterogeneity that was seen. According to the random effects model, the average effect size of the studies included in the meta-analysis on vocabulary learning achievement was calculated as 0.62 (*g* = 0.62). The findings of the meta-analysis showed an increase in vocabulary learning achievement in favor of students who were involved in the learning and teaching process using Quizlet. In terms of vocabulary learning achievement, it was found that the size of effect falls in the moderate interval. This modest effect size emphasizes the nuanced role of digital tools in education, where the impact of technology is significant but not uniformly variable across settings and groups of learners. The adoption of technologically enhanced learning environments, such as the use of Quizlet, signals a broader shift towards digital literacy and its integration into educational frameworks. Also, this effect can be explained by the rapid adaptation of students to technological integration and the effective use of innovative and technological learning methods in the classroom teaching process. When it comes to learning vocabulary in a foreign language and the number of words to be learned in a foreign language is high, it is thought that technological applications provide more effective learning. Chen et al. (2021) express that integrating educational games into language education is effective in improving students’ vocabulary acquisition, aligning with the idea of a broader shift towards digital literacy. In addition, Dewi (2023) highlights the modern shift towards the use of Quizlet for vocabulary learning, suggesting that the integration of digital tools into educational frameworks is important. The portability of laptops and smartphones has prompted the development of novel instructional methods that are thought to improve English language proficiency especially vocabulary learning (Chaikovska and Zbaravska, 2020). With the help of this process, associating target words with visuals such as various graphics, pictures, images, cartoons, etc. is thought to benefit vocabulary learning by improving the cerebral schemas of foreign language vocabulary learners. Chaikovska and Zbaravska (2020) also state that associating unknown words with visuals, such as bright graphics and exaggerated pictures, benefits vocabulary acquisition. According to Andarab (2019), using technology to contextualize vocabulary items during vocabulary acquisition improves the vocabulary learning process. This reinforcement of learning through visual aids and contextualization aligns with cognitive theories of multimedia learning, which posit that learners can more effectively process and retain information when it is presented in both verbal and visual formats. By suggesting that visual context plays an important role in memory and learning, it is suggested that visual context directs spatial attention, helps to realize implicit learning and can increase memory retention (Chun and Jiang, 1998). The findings also highlight Quizlet’s usefulness in vocabulary learning and highlight the need of utilizing Quizlet to contextualize lexical items in collocations to improve vocabulary acquisition. Chaikovska and Zbaravska (2020), while explaining the effect of Quizlet application on vocabulary learning, emphasized that the graphic presentation of the words in the program sets enriches cognitive visualization and can increase the level of word memorization by using the potential of the right hemisphere of the learners’ brain. In his study, Sanosi (2018) also highlights Quizlet’s potential in improving vocabulary learning. Sanosi (2018) asserts that Quizlet’s effectiveness as an e-learning tool for enhancing vocabulary acquisition can be linked to the increasing influence of information technology in many facets of life. The majority of daily duties for the younger generation of learners are completed on smart devices that are connected to the internet. Quizlet’s incorporation into daily technology use illustrates how learning and technology may coexist together and shows how relevant it is to the modern student’s digital habits. The principles of timed repetition and active recall, which support effective learning processes, form the foundation of Quizlet’s cognitive engagement, which is facilitated through repeated exploration and interactive quizzes.

The second research question addressed in this study focused on the effectiveness of the Quizlet on students’ vocabulary learning retention. A meta-analysis including five relevant research was carried out to provide an answer to this query. A homogeneity test was performed to determine the suitability of employing either the fixed effects model or the random effects model in the research. The results of the homogeneity test indicated a statistically significant difference, signifying a heterogeneous distribution of effect sizes among the studies (*Q* = 20.997; *p* < 0.05). The mean of the effect sizes was calculated as 0.74 (*g* = 0.74). It was determined that the *I*^2^ value obtained in the study showed heterogeneity with 80.947%. Since there was heterogeneity among the studies, effect sizes were calculated with the random effects model. According to the research findings, the average effect size indicated a moderate level of effectiveness in favor of the Quizlet. Quizlet has a moderate impact on vocabulary retention for those learning a foreign language. This impact can be attributed to several factors. The positive impact of Quizlet on retention can be attributed to the testing effect, which suggests that the act of quizzing helps learners identify knowledge gaps and actively seek out new information (Karpicke and Roediger, 2008; Nguyen and Van Le, 2022). This finding illuminates the importance of Quizlet’s interactive features for retention of learnt information and the importance of engaging in retrieval practice. The theoretical basis for this effect can be attributed to the test effect, which suggests that retrieval practice increases long-term memory retention by engaging in continuous repetition. This principle underlines the importance of incorporating active retrieval techniques into learning strategies, especially for vocabulary retention. This theory is supported by research indicating that repeated testing produces a significant positive effect on delayed recall, compared to repeated studying after learning (Karpicke and Roediger, 2008). Additionally, Nguyen et al. (2022) expressed that Quizlet creates motivation and arouses interest in learning within students’ consciousness, contributing to its impact on vocabulary retention. Moreover, the convenience and effectiveness of Quizlet, along with its features designed to be fun, make it appealing to students, thereby positively influencing vocabulary retention (Aprilani and Suryaman, 2021; Pham, 2022). The fun and motivational aspects of Quizlet, along with its interactive and engaging design, support intrinsic motivation theories that emphasize the role of enjoyment and interest in sustaining learning engagement, achievement and retention. The impact of Quizlet on vocabulary retention can also be attributed to its ability to address prevalent problems among learners in the digital era, such as low participation and difficulties in maintaining learners’ attention to lessons (Anjaniputra and Salsabila, 2018). Moreover, the incorporation of integrated skills and cognitive visualization in Quizlet makes it a useful ICT tool in vocabulary learning, contributing to its impact on vocabulary retention (Chaikovska and Zbaravska, 2020). Dual coding theory, which enhances vocabulary retention by activating both verbal and nonverbal systems, is responsible for Quizlet’s effect on students’ success in vocabulary learning and retention (Körlü and Mede, 2018). Quizlet’s effectiveness in increasing vocabulary retention through dual coding theory is thought to promote more robust encoding and retrieval of vocabulary by enhancing and strengthening the synergistic interaction between linguistic and visual information processing. Furthermore, it has been determined that Quizlet’s influence on students’ performance in vocabulary learning and retention is partially due to the dual coding theory (DCT) (Körlü and Mede, 2018). According to the study’s participants, utilizing Quizlet gave them confidence that they had learned the vocabulary items and helped them rapidly and easily remember the new words they had learnt thanks to its study and fun aspects (Körlü and Mede, 2018). This confidence and ease of recall provided by Quizlet can not only make and enhance the learning experience more effective, but can also contribute to a positive learning environment, in line with self-efficacy theory, which suggests that belief in one’s ability to succeed in certain situations or perform a task can significantly influence learning outcomes (Laufer and Hulstijn, 2001). The application of Quizlet has been found to contribute to the development of linguistic intelligence, enriching students’ vocabulary banks and enhancing their vocabulary mastery, thereby impacting vocabulary retention (Lubis et al., 2022). The use of Quizlet as a learning resource has also been recognized as an effort to develop the digital literacy of learners and motivate them to learn, further contributing to its impact on vocabulary retention (Setiawan and Putro, 2021). The role of Quizlet in advancing digital literacy and linguistic intelligence underlines the multifaceted benefits of digital learning tools, suggesting that their impact extends beyond immediate learning outcomes to include broader educational and developmental gains. Research on the impact of visual features on vocabulary learning and retention (Hashemi and Pourgharib, 2013) may provide an explanation for this discrepancy as it suggests that incorporating visual aids helps students remember and retain words more readily. On the contrary, there is also a study that supports that Quizlet is less effective in retention than traditional paper flashcards. In the study by Ashcroft et al. (2018), the findings indicate that for delayed gains, there is an even stronger negative association between proficiency and Quizlet’s improved performance compared to paper flashcards. In actuality, advanced individuals lost their digital gains much more quickly than they did their paper gains. This contrast provides an opportunity to further investigate the conditions under which digital tools such as Quizlet optimize learning outcomes and highlights the importance of personalized and adaptive learning approaches that address learners’ different needs and proficiency levels.

The last problem of this study was to investigate the effectiveness of Quizlet on students’ attitude based on the findings of the studies. To find an answer to this problem, a meta-analysis of 2 studies was conducted. Homogeneity test was conducted to determine whether it is appropriate to use the fixed effects model or the random effects model in the research. According to the results of the homogeneity test, no difference was found, and it was concluded that the effect size distribution of the studies was homogeneous (*Q* = 2.003; *p* > 0.05). It was determined that the *I*^2^ value obtained in the study showed homogeneity with 50.081%. Since there was homogeneity among the studies, effect sizes were calculated with the fixed effects model. According to the fixed effects model, the average effect size of the studies included in the meta-analysis attitude was calculated as 0.37 (*g* = 0.37). According to the research findings, the mean effect size value was found to be positive. The average effect size indicated a small level of effectiveness. This small but positive effect on attitudes towards language learning with Quizlet can be explained by the fact that digital tools can increase student engagement and motivation, but the magnitude of this effect can vary depending on factors such as implementation processes, student preferences and educational context. This variability requires a careful and detailed understanding of how digital tools such as Quizlet fit into the wider educational system and their role in shaping student attitudes and motivation. This also can be explained by the lack of quantitative studies measuring student attitudes towards the Quizlet application. The paucity of limited quantitative research on student attitudes towards Quizlet underlines the need for more robust empirical research that can offer deeper insights into how digital tools influence students’ psychological and emotional engagement in language learning. The lack of quantitative studies measuring student attitudes towards the Quizlet application is further supported by (Mykytka, 2023), who highlighted the focus on vocabulary acquisition and the limited investigation of the effect of Quizlet on other skills. On the other hand, there are many qualitative studies proving that the Quizlet application increases student attitude and motivation positively (Chien, 2015; Dizon, 2016; Alastuey and Nemeth, 2020; Setiawan and Wiedarti, 2020; Lubis et al., 2022; Nguyen et al., 2022; Pham, 2022; Zeitlin and Sadhak, 2022). Qualitative studies supporting the positive impact of Quizlet on student attitude and motivation emphasize the subjective and experiential dimensions of learning with digital tools and reveal that the effectiveness of these tools can be significantly affected by students’ perceptions and experiences. The effectiveness of Quizlet in increasing students’ attitude and motivation towards learning has been widely documented in the literature. The reasons for this positive influence can be attributed to various factors. Firstly, Quizlet has been shown to create a motivational learning environment by making the process of vocabulary acquisition more enjoyable and engaging for students and thus having positive impact on attitude (Alastuey and Nemeth, 2020; Chaikovska and Zbaravska, 2020; Setiawan and Wiedarti, 2020; Aprilani and Suryaman, 2021; Berliani and Katemba, 2021; Mykytka, 2023). This enhancement of the learning environment through engagement and enjoyment reflects broader principles of educational psychology that emphasize the importance of positive emotional experiences in enhancing learning and student attitude. The interactive and gamified nature of Quizlet, such as its flashcards, quizzes, and other interactive activities, enhance students’ attitude, interest and intrinsic motivation in learning vocabulary (Körlü and Mede, 2018; Alastuey and Nemeth, 2020; Nguyen et al., 2022; Mykytka, 2023). The gamification of learning processes facilitated by using Quizlet is also considered to be compatible with the principles of game-based learning, which suggest that game elements can significantly increase attitude and engagement and thus positively affect learning outcomes. Additionally, the convenience and effectiveness of Quizlet have been reported to positively influence students’ attitude, motivation and interest in vocabulary learning (Pham, 2022; Zeitlin and Sadhak, 2022). This positive effect of Quizlet on student attitude and achievement demonstrates the importance of the role of user-friendly and effective digital tools in increasing student engagement and satisfaction in the learning process. And, Quizlet app’s wide range of activities and high degree of instant feedback, which paper flashcards could not match, may have increased and maintained students’ attitude engagement and motivation (Ashcroft et al., 2018). In contrast to traditional learning methods, the immediate and fast feedback provided by Quizlet facilitates more effective learning by helping students to quickly identify and correct errors, which can be explained by the feedback loop theory, which suggests that timely and relevant feedback is crucial for learning, engagement and attitude. This theory is supported by the research which discusses the immediate feedback assessment technique and its role in promoting learning and correcting inaccurate first responses (Epstein et al., 2002; Dihoff et al., 2004).

The current study demonstrates that Quizlet is an appropriate educational technology for fostering the learners’ cognitive and emotional dimensions in foreign learning process, especially in vocabulary learning. Apart from the technology tools and programs that aid in language acquisition, Quizlet has become a highly effective instrument for improving vocabulary knowledge and fostering learners’ attitude. After taking into account all of its benefits, Quizlet is an efficient web-based mobile learning tool that is entertaining, motivating, and helpful for learning vocabulary. Also, the design of the Quizlet application is suitable for autonomous learners, which enhances students’ attitude and motivation in learning vocabulary (Setiawan and Wiedarti, 2020). This availability for autonomous learning reflects the growing demand for personalized and self-directed learning opportunities in modern education and highlights Quizlet’s role in meeting these evolving educational needs. The literature indicates that students’ opinions of Quizlet as a tool for vocabulary development were important in determining whether or not language learners should utilize it to increase their vocabulary. The importance and evaluation of learner views emphasizes the importance of learner-centered approaches in educational technology research and shows that the effectiveness of digital tools such as Quizlet can be significantly affected by learners’ achievement, learning retention, attitudes. Overall, the literature supports the importance of Quizlet in enhancing learners’ vocabulary learning, retention, and attitude, making it a valuable tool for educators and learners alike. This comprehensive perspective emphasizes the multifaceted impact of Quizlet on language learning, from learning achievement and retention to student attitudes, and underlines the need for its integration into language teaching practices. The study’s overall conclusions highlight the importance of incorporating mobile assisted language learning and teaching resources like Quizlet into teaching and learning practices and curricula. Quizlet was found to be a useful and effective application that supports students’ performance and autonomy in vocabulary learning by giving them increased exposure to the target words through a variety of functions and a study environment that is like a game. It is thought that the integration of Quizlet into language teaching, especially vocabulary teaching, could mean a shift towards more interactive, engaging and learner-centered approaches, reflecting the ongoing evolution of educational paradigms in the digital age.

## 6 Conclusion

This meta-analysis was conducted to investigate the effects of Quizlet on the cognitive and affective outcomes of foreign language learners, especially on vocabulary learning achievement, retention and attitude, and to reach a general conclusion on this issue. The findings of this study suggest that Quizlet has a moderate positive effect on vocabulary learning and retention and a small effect on learner attitude, suggesting that Quizlet may have the potential to be a valuable educational technology tool in language acquisition. The integration of Quizlet into the learning process not only facilitates students’ adaptation to modern technological tools, but also enhances their ability to effectively learn and retain large amounts of foreign vocabulary knowledge. This effectiveness can be partly attributed to Quizlet’s combination of visual aids and cognitive visualization techniques, which, as many studies have shown, play a crucial role in enhancing vocabulary acquisition and aiding retention. These visual components, coupled with the interactive nature of Quizlet, can help create a more engaging and immersive learning environment, which can be especially useful in the context of foreign language acquisition, especially in vocabulary learning, where visual associations can significantly support the learning process.

Despite these positive findings, our analysis also indicates a relatively smaller effect of Quizlet on learners’ attitudes towards vocabulary learning. This may be attributed to the limited number of quantitative studies specifically addressing this aspect of language learning. While existing qualitative research provides some evidence of Quizlet’s positive impact on learner motivation and attitude, there is a clear need for more rigorous, quantitative investigations to substantiate these observations. The discrepancy between qualitative and quantitative findings highlights a potential gap in the research, suggesting that future studies should aim to quantitatively measure and understand the nuances of how digital learning tools like Quizlet influence learners’ attitudes. Thanks to technological advances and the latest educational techniques, teachers are now able to use a wide range of online and mobile applications. The nuanced relationship between technology use and student attitude raises important questions about the adaptability and effectiveness of digital tools in different learning contexts. As digital learning environments continue to evolve, understanding these dynamics becomes increasingly important to optimize their design and implementation for maximum educational benefit. In light of these findings, it is clear that Quizlet shows considerable promise in improving vocabulary learning and retention but that further research is needed to fully understand its impact on student attitudes. Such insights can be important for educators and curriculum designers to make informed decisions about integrating digital tools such as Quizlet into language learning programs. The future of language education increasingly involves the integration of technology into educational processes. It is of utmost importance that such endeavors are developed and channeled in such a way that this integration contributes positively to student learning, achievement, attitudes and many other aspects.

In conclusion, while our study confirms the effectiveness of Quizlet in improving vocabulary learning, retention and learner attitude, it also highlights areas where further research is needed. Quizlet, with its interactive and engaging features, emerges as a valuable tool that facilitates the learning process and is effective and helpful in the learning processes of language educators and learners. It is thought that it may be important to integrate mobile assisted language learning resources such as Quizlet into educational practices and curricula as auxiliary tools to support foreign language learners’ performance in vocabulary acquisition and to improve their attitudes positively. This study may shed light and open new avenues for future research on how different aspects of Quizlet and similar platforms can be optimized for educational success.

## 7 Limitations and future research directions

### 7.1 Quantitative research on learner attitudes

A significant gap has been identified in quantitative research addressing the impact of Quizlet on students’ attitudes. Future studies should utilize robust quantitative methodologies to systematically assess how Quizlet affects student attitudes.

### 7.2 Temporal scope of studies

Longitudinal studies are recommended to examine the sustained impact of Quizlet on vocabulary learning over long periods of time. Such studies would provide invaluable insights into the long-term effectiveness and adaptability of Quizlet in evolving educational settings.

### 7.3 Comparative efficacy studies

Future research should take a more comprehensive approach, including a wider range of comparative analyses between Quizlet and both traditional and digital learning tools. Such comparative studies would deepen our understanding of Quizlet’s effectiveness relative to other methods and provide detailed insights into Quizlet’s unique advantages and areas for improvement.

### 7.4 Contextual diversity in learning environments

The majority of the studies reviewed focused on formal educational settings. There is a need for research exploring the use of Quizlet in more varied contexts, including informal learning environments and self-directed learning scenarios. Investigating Quizlet’s application in these diverse settings would offer a more holistic understanding of its adaptability and effectiveness across different learning modalities.

### 7.5 Technological advancements and evolving educational technologies

The rapid development of technology and its integration into educational contexts requires the continuous evaluation of tools such as Quizlet. Future studies should not only focus on Quizlet’s current functionalities, but also consider emerging technological developments that may affect its educational utility. Continuous evaluation is crucial to understand how evolving features and changes in user interaction with technology affect learning outcomes.

## Data availability statement

The original contributions presented in the study are included in the article/supplementary material, further inquiries can be directed to the corresponding author.

## Author contributions

OÖ: Writing – original draft. HS: Writing – review & editing.
